# FGF1 protects neuroblastoma SH-SY5Y cells from p53-dependent apoptosis through an intracrine pathway regulated by FGF1 phosphorylation

**DOI:** 10.1038/cddis.2017.404

**Published:** 2017-08-31

**Authors:** Caroline Pirou, Fatemeh Montazer-Torbati, Nadège Jah, Elisabeth Delmas, Christelle Lasbleiz, Bernard Mignotte, Flore Renaud

**Affiliations:** 1 Laboratoire de Génétique et Biologie Cellulaire, EA4589, Université de Versailles Saint-Quentin-en-Yvelines (UVSQ), Université Paris-Saclay, École Pratique des Hautes Etudes (EPHE), PSL Research University, 2 Avenue de la Source de la Bièvre, Montigny-Le-Bretonneux 78180, France

## Abstract

Neuroblastoma, a sympathetic nervous system tumor, accounts for 15% of cancer deaths in children. In contrast to most human tumors, *p53* is rarely mutated in human primary neuroblastoma, suggesting impaired p53 activation in neuroblastoma. Various studies have shown correlations between *fgf1* expression levels and both prognosis severity and tumor chemoresistance. As we previously showed that fibroblast growth factor 1 (FGF1) inhibited p53-dependent apoptosis in neuron-like PC12 cells, we initiated the study of the interaction between the FGF1 and p53 pathways in neuroblastoma. We focused on the activity of either extracellular FGF1 by adding recombinant rFGF1 in media, or of intracellular FGF1 by overexpression in human SH-SY5Y and mouse N2a neuroblastoma cell lines. In both cell lines, the genotoxic drug etoposide induced a classical mitochondrial p53-dependent apoptosis. FGF1 was able to inhibit p53-dependent apoptosis upstream of mitochondrial events in SH-SY5Y cells by both extracellular and intracellular pathways. Both rFGF1 addition and etoposide treatment increased *fgf1* expression in SH-SY5Y cells. Conversely, rFGF1 or overexpressed FGF1 had no effect on p53-dependent apoptosis and *fgf1* expression in neuroblastoma N2a cells. Using different FGF1 mutants (that is, FGF1^K132E^, FGF1^S130A^ and FGF1^S130D^), we further showed that the C-terminal domain and phosphorylation of FGF1 regulate its intracrine anti-apoptotic activity in neuroblastoma SH-SY5Y cells. This study provides the first evidence for a role of an intracrine growth factor pathway on p53-dependent apoptosis in neuroblastoma, and could lead to the identification of key regulators involved in neuroblastoma tumor progression and chemoresistance.

The fibroblast growth factor 1 (FGF1) is an oncogene, which regulates many cellular processes including cell proliferation, differentiation and survival.^1, 2, 3^ FGF1 has been linked to tumor development, as it is upregulated in various cancers (breast, ovarian, gliomas and astrocytomas). Correlation between *fgf1* expression, prognosis severity and tumor chemoresistance has been found.^4, 5, 6, 7^

FGF1 is highly expressed in central and peripheral nervous systems and is involved in neural development.^1, 8, 9, 10^ FGF1 neurotrophic and anti-apoptotic activities are well documented both *in vitro* and *in vivo.*^3, 11, 12, 13, 14, 15^ However, the mechanism of action of the FGF1 anti-apoptotic activity is far from being fully elucidated and the putative role of FGF1 in the progression and chemoresistance of neuronal tumors remains to be characterized.

In the neuronal-like PC12 cells, we previously showed that FGF1, which lacks a secretion peptide signal but contains a nuclear localization sequence (NLS), acts by an intracrine and nuclear pathway for both its neurotrophic activity and its p53-dependent cell death protection. We also showed that FGF1 anti-apoptotic activity could be regulated by phosphorylation.^3, 14, 15^

Neuroblastoma is a tumor of the sympathetic nervous system. It is the most common extracranial solid tumor and accounts for 15% of cancer deaths in young children.^16^ In contrast to most human tumors, human primary neuroblastoma are rarely mutated in *p53*,^17^ suggesting that the p53 oncosuppressive signaling cascade must be bypassed in these tumors by blocking the upstream activation of p53 (that is, by amplification of *MDM2* or hypermethylation of *p14*^*ARF*^).^18^ In most neuroblastoma cell lines, p53-dependent apoptosis can be induced by genotoxic or oxidative stress and/or by inhibition of survival growth factor pathways.^19, 20, 21, 22, 23, 24^ In these cells, genotoxic stress induces the classical mitochondrial apoptotic pathway as well as the death-receptor extrinsic apoptotic pathway.

In neuroblastoma tumors, overexpression of survival factors and/or their receptors (that is, BDNF, TrK-B, IGF-1R) were detected, which could regulate the apoptotic process whether p53-dependent or not.^18, 24, 25^ However, only few studies examined growth factor anti-apoptotic activities in neuroblastoma cell lines. BDNF and IGF-I protected human neuroblastoma SH-SY5Y or SH-EP1 cells from chemotherapeutic drug-induced apoptosis through the PI3K/AKT pathway,^26, 27, 28^ FGF2 and FGF4 impaired oxidative stress induced-apoptosis, and FGF1 inhibited serum depletion induced-apoptosis in SH-SY5Y cells.^13, 29^ To our knowledge, no study was performed to examine the role of growth factors on p53-dependent apoptosis in neuroblastoma.

In the present study, we examined the activity of FGF1 on the p53-dependent apoptotic pathway in neuroblastoma cell lines. In both human SH-SY5Y and murine N2a cells, we showed that etoposide induced a classical mitochondrial p53-dependent apoptosis. FGF1 inhibited p53-dependent apoptosis upstream mitochondrial events in SH-SY5Y cells by extracellular and intracellular pathways. Conversely, it had no effect in N2a cells. Using FGF1^K132E^, FGF1^S130A^ and FGF1^S130D^ mutants, we further showed that both the C-terminal domain and phosphorylation of FGF1 regulate its intracrine anti-apoptotic activity in SH-SY5Y cells.

## Results

### Extracellular FGF1 protects SH-SY5Y cells but not N2a cells from p53-dependent apoptosis

**Figure 1:**
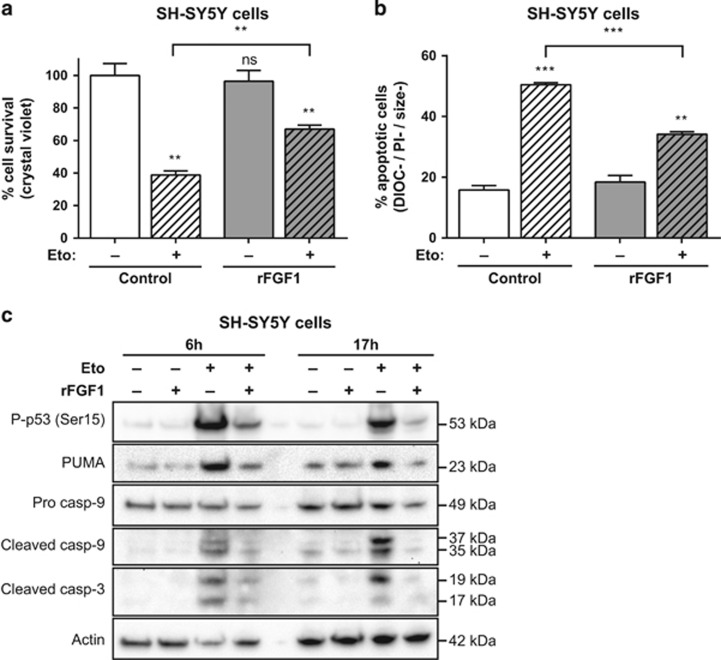
Extracellular FGF1 protects SH-SY5Y cells from p53-dependent apoptosis. (**a**) SH-SY5Y cells were pretreated or not by adding recombinant FGF1 and heparin for 48 h (rFGF1) in the culture medium, then cells were treated or not with etoposide for 24 h (Eto). Cell survival was analyzed by crystal violet nuclei staining. (**b**) Following the same treatments, SH-SY5Y apoptotic cells were characterized by flow cytometry after DiOC_6_(3) and PI staining. Apoptotic cells correspond to the low DiOC_6_(3) (low ΔΨm, noted DIOC−) and low PI (to exclude necrotic cells, noted PI−) staining and small-sized cells (a hallmark of apoptotic cell condensation, noted size−). For (**a** and **b**), the graphs represent the mean±S.E.M. of three independent experiments. Student’s *t*-tests were performed relative to the control cells, except where indicated (*n*=3; n.s.: *P*>0.05; **P⩽0.01; ***P⩽ 0.001). (**c**) SH-SY5Y cells were pretreated or not with recombinant FGF1 (rFGF1) for 48 h, and then treated or not with etoposide (Eto) for 6 h or 17 h. Twenty micrograms of the corresponding cell lysate proteins were used to analyze by western blot the levels of P-p53 (Ser15) that reveals p53 activation, of the p53 proapoptotic target PUMA, of pro- and cleaved caspase-9 forms and cleaved caspase-3. Actin detection was used as a control

**Figure 2:**
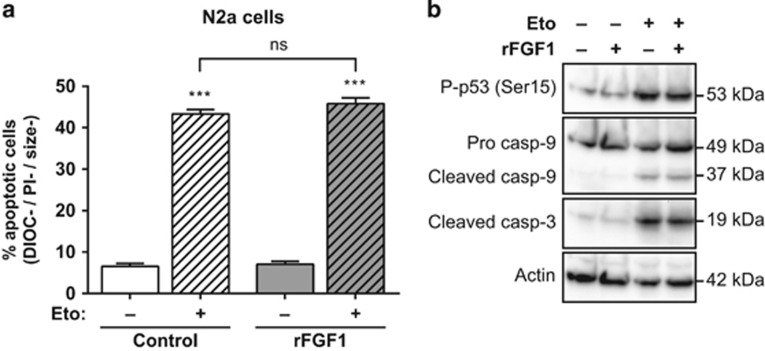
Extracellular FGF1 does not protect N2a cells from p53-dependent apoptosis. (**a**) N2a cells were pretreated or not by adding recombinant FGF1 and heparin in the culture medium (rFGF1) for 48 h, then treated or not with etoposide (Eto) for 24 h. N2a apoptotic cells were characterized by flow cytometry after DiOC_6_(3) and PI staining (apoptotic cells are the DIOC−, PI− and size− cells). The graph represents the mean±S.E.M. of three independent experiments. Student’s *t*-tests were performed relative to control cells, except where indicated (*n*=3; n.s.: *P*>0.05; ***: *P*⩽0.001). (**b**) N2a cells were pretreated or not with recombinant FGF1 (rFGF1) for 48 h, and then treated or not with etoposide (Eto) for 24 h. Twenty micrograms of the corresponding cell lysate proteins were used to analyze by western blot the levels of P-p53 (Ser15) that reveals p53 activation, of pro- and cleaved caspase-9 forms and cleaved caspase-3. Actin detection was used as a control

To test the putative protective activity of FGF1 on p53-dependent cell death in neuroblastoma models, we examined the effect of adding recombinant FGF1 (rFGF1) in the culture medium on SH-SY5Y and N2a cell lines after etoposide treatment. Etoposide inhibits topoisomerase II, which induces p53-dependent apoptosis in SH-SY5Y cells.^22^ After 48 h of pretreatment with rFGF1, SH-SY5Y and N2a cells were treated with etoposide with or without rFGF1 for 24 h. Cell death of rFGF1- and etoposide-treated SH-SY5Y and N2a cells was then analyzed (Figures 1 and 2).

In SH-SY5Y cells, etoposide induces cell death as assessed by the crystal violet nuclei staining method (Figure 1a). The survival rate decreased down to 40% after 24 h of etoposide treatment. After 48 h of rFGF1-pretreatment followed by a 24 h etoposide and rFGF1 treatment, SH-SY5Y cell survival increased to 70%. While rFGF1 pretreatment and treatment did not modify cell survival in the absence of etoposide, addition of rFGF1 protected SH-SY5Y from etoposide-induced cell death.

To further characterize the cell death process, SH-SY5Y treated with etoposide in the absence or presence of rFGF1 were analyzed by flow cytometry after DiOC_6_(3) and propidium iodide (PI) staining. After 24 h of etoposide treatment, 50% of SH-SY5Y cells were apoptotic (Figure 1b). Addition of rFGF1 reduced the rate of etoposide-induced SH-SY5Y apoptotic cells, 55% of protection was detected. rFGF1 alone had no effect on cell viability. Etoposide induced the intrinsic apoptotic pathway in SH-SY5Y and rFGF1 protected these cells from this apoptosis upstream of mitochondrial events.

Various markers of p53-dependent apoptosis were then analyzed by western blot in rFGF1- and etoposide-treated SH-SY5Y cells (Figure 1c). The phospho-p53 (Ser15) form that reveals p53 activation, the pro-apoptotic BH3-only protein p53-upregulated modulator of apoptosis (PUMA) encoded by a transcriptional target gene of p53, and the cleavage of both caspase-9 and -3 were analyzed. After 6 or 17 h of etoposide treatment, SH-SY5Y cells showed a marked increase of P-p53 (Ser15), PUMA and cleaved forms of caspase-9 and -3. These results confirmed that etoposide induces p53-dependent apoptosis in SH-SY5Y cells. Addition of rFGF1 decreases the levels of all these markers, indicating that rFGF1 decreases the etoposide-induced activation of p53, the transactivation of its target PUMA and the activation of both caspase-9 and -3.

In N2a cells, we also examined the activity of rFGF1 on etoposide-induced cell death by flow cytometry after DiOC_6_(3) and PI staining (Figure 2a). After 24 h of etoposide-treatment, ~45% of apoptotic N2a cells were detected. However, the addition of rFGF1 did not modulate the rate of apoptotic N2a cells. We further showed by western blotting that etoposide increased the levels of both p53 phosphorylation and caspase cleavage, while rFGF1 did not regulate these events in N2a cells (Figure 2b).

Therefore, extracellular FGF1 protects human neuroblastoma SH-SY5Y cells from p53-dependent apoptosis upstream mitochondrial events by decreasing p53 phosphorylation, *PUMA* transactivation and caspase activation. By contrast, extracellular FGF1 does not protect mouse neuroblastoma N2a cells from p53-dependent apoptosis.

### Extracellular FGF1 and etoposide increase FGF1 endogenous expression in SH-SY5Y cells, in contrast to N2a cells

Addition of rFGF1 protected SH-SY5Y cells from p53-induced apoptosis (Figures 1a–c); however, an rFGF1-pretreatment of at least 24 h is required to detect this protection. In PC12 cells, we have previously shown that extracellular FGF1 induces the expression of endogenous *fgf1* and that intracellular FGF1 protects these cells from p53-dependent apoptosis.^3, 14^ In SH-SY5Y cells, rFGF1 addition was shown to increase *fgf1* expression in the absence of serum, and FGF1 overexpression was shown to protect these cells from serum depletion-induced cell death.^13^

**Figure 3:**
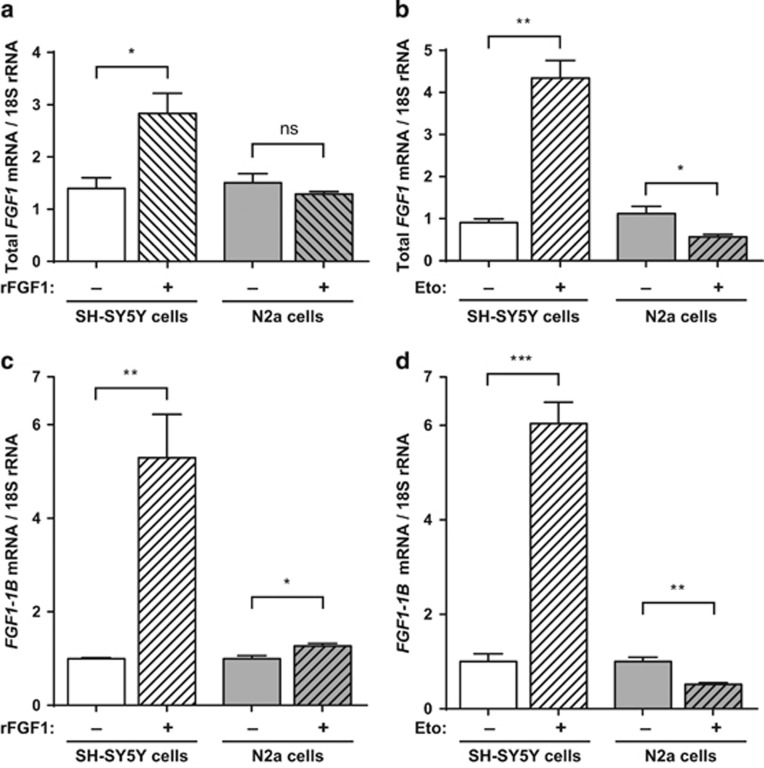
Extracellular FGF1 and etoposide increase endogenous *fgf1* expression in SH-SY5Y cells, in contrast to N2a cells. SH-SY5Y and N2a cells were treated or not with rFGF1 for 72 h (**a**–**c**) or etoposide for 16 h (**b**–**d**). The levels of all *fgf1* mRNAs (**a**,**b**) or of the alternative 1B *fgf1* mRNA (**c**,**d**) were analyzed by RT-PCR. The 18S rRNA levels were used as a control for quantifications. The graphs represent the mean ±S.E.M. of three independent experiments. Student’s *t*-tests were performed (*n*=3; n.s.: *P*>0.05; *P⩽0.05; **P⩽0.01; ***P⩽0.001)

Therefore, we examined by RT-PCR the regulation of *fgf1* expression induced by rFGF1- or etoposide-treatment in the presence of serum in SH-SY5Y and N2a cells. After 3 days of rFGF1 treatment, a two-fold increase of *fgf1* mRNA levels was detected in SH-SY5Y cells. No similar regulation was detected in N2a cells (Figure 3a). After 16 h of etoposide treatment, a four-fold increase of *fgf1* mRNA was detected in SH-SY5Y cells, while a two-fold decrease of *fgf1* mRNA was detected in N2a cells (Figure 3b). Etoposide treatment upregulates endogenous *fgf1* expression in SH-SY5Y but downregulates *fgf1* expression in N2a cells.

*fgf1* expression can be initiated by four alternative promoters, which permit the synthesis of different *fgf1* transcripts containing 5′UTR alternative sequences (1A to 1D).^1^ Using specific primers, we showed by RT-PCR that the 1B *fgf1* mRNA is the major transcript detected in SH-SY5Y and N2a cells (Figures 3c and d). Nevertheless, the other *fgf1* mRNAs (that is, 1A and 1D mRNAs in SH-SY5Y cells and 1A, 1C and 1D mRNAs in N2a cells) could also be detected at lower levels. All *fgf1* mRNAs were regulated by rFGF1 and/or etoposide in these cells, although not always similarly (Supplementary Figure 1).

### FGF1 overexpression protects SH-SY5Y cells but not N2a cells from p53-dependent apoptosis

The study of rFGF1 activity on both p53-induced cell death and *fgf1* expression in both neuroblastoma cell lines suggests that the protective activity of extracellular FGF1 on p53-dependent apoptosis in SH-SY5Y could be mediated by endogenous FGF1. To test this hypothesis, we examined the effects of intracellular FGF1 on p53-dependent apoptosis in both cell lines.

**Figure 4:**
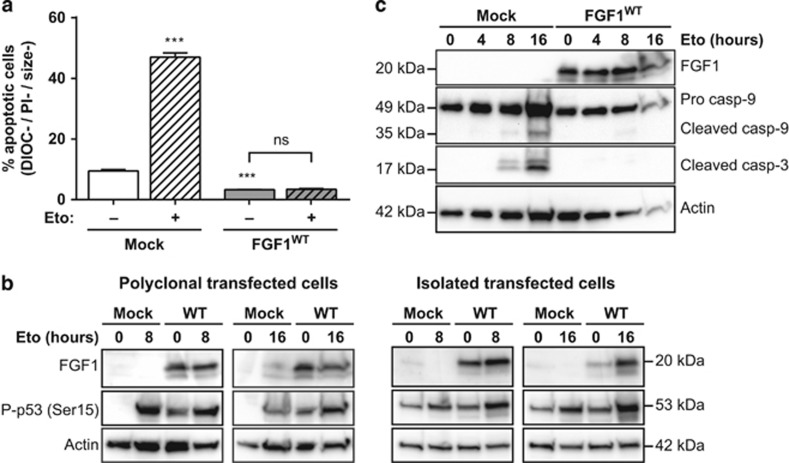
Intracellular FGF1^WT^ protects SH-SY5Y cells from p53-dependent apoptosis. Stably transfected SH-SY5Y cells with either FGF1^WT^ or empty (mock) expression vectors were treated or not with etoposide for 16 h. (**a**) Polyclonal transfected SH-SY5Y apoptotic cells were characterized by flow cytometry after DiOC_6_(3) and PI staining (apoptotic cells are the DIOC−, PI− and size− cells). The graph represents the mean±S.E.M. of three independent experiments. Student’s *t*-tests were performed relative to the mock control, except where indicated (*n*=3; n.s.: *P*>0.05; ***: *P*⩽0.001). (**b**) FGF1 expression and p53 activation (phosphorylated Ser15) were assessed by western blot in either polyclonal transfected cells (left panel) or isolated transfected cell lines (right panel). Actin detection was used as control. (**c**) FGF1 expression, caspase-9 and -3 cleavages were assessed in polyclonal transfected cells by western blot. Actin detection was used as control

To investigate the role of intracellular FGF1, SH-SY5Y cells were stably transfected with an FGF1^WT^ expression vector to overexpress intracellular FGF1^WT^ or with an empty expression vector (mock) as a control. Geneticin-resistant polyclonal transfected SH-SY5Y cells were then treated with etoposide for 16 h, and the percentage of apoptotic cells was quantified by flow cytometry after DIOC_6_(3) and PI staining (Figure 4a). After etoposide treatment, less than 5% of FGF1^WT^-transfected cells displayed an apoptotic phenotype, while 50% of mock-transfected cells were apoptotic. Therefore, overexpressing FGF1^WT^ abolished the etoposide-induced apoptosis.

We then examined by western blot the levels of FGF1, p53 phosphorylation and caspase cleavage in FGF1^WT^ and mock transfected SH-SY5Y cells after 0, 4, 8 or 16 h of etoposide treatment (Figures 4b and c). In FGF1^WT^ transfected SH-SY5Y cells, a high level of FGF1 was detected as a result of the transfection. After etoposide treatment, the levels of p53 phosphorylation and cleaved caspase-9 and -3 increased in mock-transfected SH-SY5Y cells. In *fgf1* overexpressing cells, etoposide-induced caspase cleavage was inhibited, according to the inhibition of the apoptotic process. However, FGF1 did not inhibit p53 phosphorylation in *fgf1* overexpressing cells, either in polyclonal or isolated stable transfected cells. Therefore, FGF1^WT^ overexpression inhibits the etoposide-induced caspase cleavage and apoptosis in SH-SY5Y neuroblastoma cells, without regulating p53 phosphorylation.

**Figure 5:**
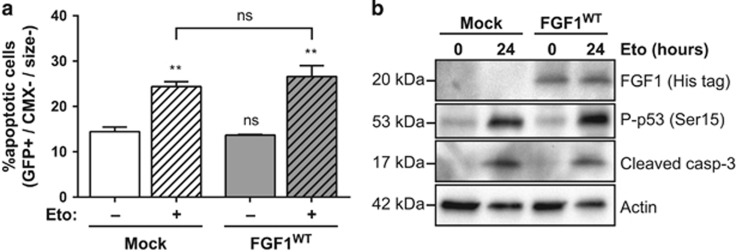
Intracellular FGF1 does not protect N2a cells from p53-dependent apoptosis. (**a**) N2a cells were transiently co-transfected with GFP and FGF1^WT^ or empty (mock) expression vectors and then treated or not with etoposide for 24 h. Following transfection and treatment, N2a transfected apoptotic cells were quantified by flow cytometry after CMX-Ros staining. Transfected apoptotic cells correspond to the high GFP (transfected cells, GFP+), low CMX-Ros (low ΔΨm, CMX−) and small-sized (size−) cells. The graph represents the mean±S.E.M. of three independent experiments. Student’s *t*-tests were performed relative to the control Mock cells, except where indicated (*n*=3; n.s.: *P*>0.05; **: *P*⩽0.01). (**b**) N2a cells were transiently transfected with FGF1^WT^ or empty (mock) expression vectors and then treated or not with etoposide for 24 h. FGF1 expression, p53 activation (Ser15 phosphorylation) and caspase-3 cleavage were analyzed by western blot. Actin detection was used as a control

We also investigated the activity of intracellular FGF1 in p53-dependent apoptosis in N2a cells. For that purpose, cells were transiently co-transfected with GFP and FGF1^WT^ or mock expression vectors and then treated with etoposide for 24 h (Figure 5a). Apoptotic N2a cells were characterized by flow cytometry after CMX-Ros staining and GFP analysis. In mock and FGF1^WT^ transfected N2a cells, the percentage of transfected apoptotic cells increased after etoposide treatment at similar levels. By western blot (Figure 5b), we further showed that FGF1 overexpression is detected in FGF1^WT^ transfected N2a cells; however, similar increases of p53 phosphorylation and cleaved caspase-3 were detected in mock- and FGF1^WT^-transfected N2a cells after etoposide treatment. Overexpression of FGF1 does not protect N2a cells from p53-dependent apoptosis.

### FGF1 C-terminal domain and phosphorylation regulate its intracellular anti-apoptotic activity in SH-SY5Y cells

To progress in the characterization of FGF1 anti-apoptotic activity in human neuroblastoma, we transfected SH-SY5Y cells with different mutated forms of FGF1 (that is, FGF1^K132E^, FGF1^S130A^ and FGF1^S130D^).

The extracellular and intracellular FGF1^K132E^, initially characterized for its lower affinity to heparin, displays impaired mitogenic activity in murine fibroblasts^30^ and impaired neurotrophic and anti-apoptotic activity in PC12 cells.^3, 15^ Intracellular FGF1 could be phosphorylated in the nucleus by the Protein Kinase C delta (PKCδ) on its serine 130.^31^ The mutation of this serine into an alanine impairs FGF1 phosphorylation (FGF1^S130A^), while its mutation into an aspartate mimics constitutive FGF1 phosphorylation (FGF1^S130D^). These FGF1 mutants were previously tested in PC12 cells, only FGF1^S130A^ protects these cells from p53-dependent apoptosis.^15^

**Figure 6:**
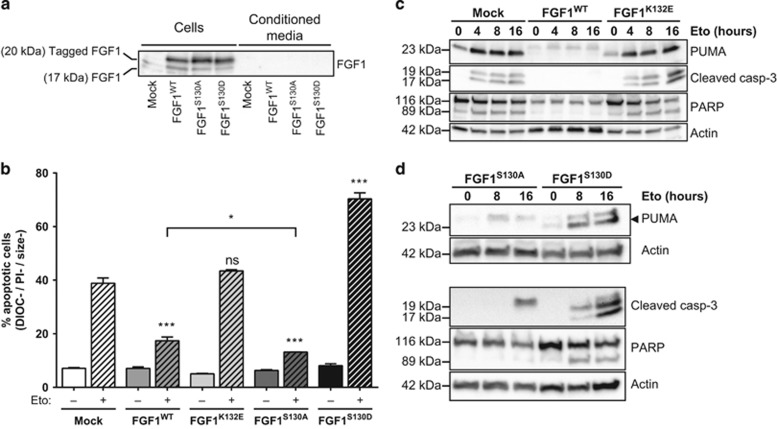
FGF1 phosphorylation inhibits its anti-apoptotic activity in SH-SY5Y cells. SH-SY5Y cells were stably transfected with FGF1^WT^, FGF1^K132E^, FGF1^S130A^, FGF^S130D^ or empty (mock) expression vectors. (**a**) FGF1 levels were examined by western blot after heparin-sepharose concentration in cell lysates and conditioned media from SH-SY5Y cells expressing FGF1^WT^, FGF1^S130A^, FGF1^S130D^ or transfected with an empty vector (mock). (**b**) Apoptosis in FGF1^WT^, FGF1^K132E^, FGF1^S130A^, FGF^S130D^ or mock stably transfected SH-SY5Y cells after 48 h of etoposide treatment was analyzed by flow cytometry after DiOC_6_(3) and PI staining. Apoptotic cells are the DIOC−, PI− and size− cells. The graph represents the mean±S.E.M. of three independent experiments. Student’s *t*-tests were performed relative to the control mock, except where indicated (*n*=3; n.s.: *P*>0.05; *: *P*⩽0.05; ***: *P*⩽0.001). (**c,d**) PUMA, cleaved caspase-3 and PARP (full-length and cleaved forms) levels were analyzed by western blot in FGF1^WT^, FGF1^K132E^, FGF1^S130A^, FGF^S130D^ or mock stably transfected SH-SY5Y cells after 0, 4, 8 or 16 h of etoposide treatment. Actin detection was used as control

We examined FGF1 levels and its intracellular *versus* extracellular localization in wild-type and mutant FGF1 transfected SH-SY5Y cells (Figure 6a). After concentration on heparin sepharose and western blot analysis, FGF1^WT^, FGF1^S130A^ and FGF1^S130D^ were detected at similar levels in transfected cell extracts but were not detected in conditioned media, thus showing that wild-type and mutant FGF1 are not secreted. It can be noticed that WT and mutant transfected FGF1 forms are V5/His-tagged (~20 kDa) and could be discriminated from endogenous FGF1 (~14–17 kDa). After heparin-sepharose, endogenous FGF1 could be detected only in cell lysates, suggesting that endogenous FGF1 is also non-secreted in neuroblastoma SH-SY5Y cells. Overexpressed FGF1^K132E^ was detected in transfected cell lysates without heparin-sepharose concentration (data not shown) but it was impossible to test its putative secretion in the conditioned medium, because of its low affinity for heparin-sepharose.

Apoptosis in FGF1^WT^, FGF1^K132E^, FGF1^S130A^, FGF^S130D^ or mock stably transfected SH-SY5Y cells after 48 h of etoposide treatment was analyzed by flow cytometry after DiOC_6_(3) and PI staining (Figure 6b). FGF1^WT^ protected SH-SY5Y cells from etoposide-induced apoptosis, while FGF1^K132E^ did not protect these cells from apoptosis. Interestingly, contrasting activities were detected for phosphorylation mutant forms of FGF1. The non-phosphorylable FGF1^S130A^ protected SH-SY5Y cells from p53-dependent apoptosis, at a significantly higher level than FGF1^WT^. By contrast, the phosphomimetic form FGF1^S130D^ sensitized SH-SY5Y cells to p53-dependent apoptosis.

Analysis of apoptotic markers in FGF1^WT^, FGF1^K132E^, FGF1^S130A^, FGF^S130D^ or mock stably transfected SH-SY5Y cells after 0, 4, 8 or 16 h of etoposide treatment was performed by western blot analysis (Figures 6c and d). Expression of FGF1^WT^ and non-phosphorylable FGF1^S130A^ inhibited the p53-induced transactivation of pro-apoptotic PUMA as well as the cleavage of both caspase-3 and its substrate PARP. By contrast, neither FGF1^K132E^ nor the phosphomimetic FGF1^S130D^ inhibited *PUMA* transactivation or caspase-3 and PARP cleavages.

These results show that the non-phosphorylable FGF1^S130A^ protects SH-SY5Y cells from p53-dependent apoptosis, in contrast to FGF1^K132E^ and FGF1^S130D^, suggesting that FGF1 phosphorylation plays a major role in inhibiting the intracrine FGF1 anti-apoptotic activity in this human neuroblastoma model.

## Discussion

**Figure 7:**
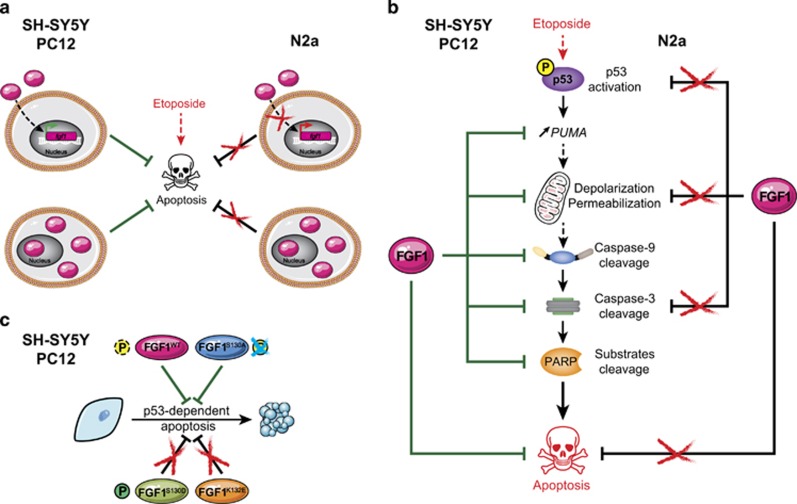
Role of extra- and intracellular FGF1 in neuronal SH-SY5Y, N2a and PC12 cells. (**a**) Both extra- and intra-cellular FGF1 protect human SH-SY5Y and rat PC12 cells from p53-dependent apoptosis (left). Endogenous *fgf1* expression is increased by rFGF1 addition (top left). In contrast, extra- and intra-cellular FGF1 show no anti-apoptotic activity, and rFGF1 addition does not increase endogenous *fgf1* expression in murine N2a cells (right). (**b**) Overexpression of intracellular FGF1 inhibits etoposide-induced apoptosis in human SH-SY5Y and rat PC12 cells by decreasing *PUMA* transactivation, mitochondrial membrane depolarization and permeabilization, and activation of caspase-9, caspase-3 as assessed by the cleavage of its substrate PARP (left). On the contrary, FGF1 overexpression does not protect N2a cells from etoposide-induced apoptosis, and modifies neither p53 activation, mitochondrial depolarization and permeabilization, nor cleavage of caspase-3 (right). (**c**) In human SH-SY5Y and rat PC12 cells, overexpression of wild-type FGF1 or non-phosphorylable FGF1^S130A^ inhibits p53-dependent apoptosis, while overexpression of the phosphomimetic FGF1^S130D^ or the FGF1^K132E^ mutant does not. Therefore, phosphorylation of FGF1 inhibits its anti-apoptotic activity in SH-SY5Y and PC12 cells. This figure compiles the results of the present study performed in neuroblastoma SH-SY5Y and N2a cells and of previous studies performed in pheochromocytoma PC12 cells^14, 15, 32^

In this study, we first showed that FGF1 inhibits p53-dependent apoptosis upstream of mitochondrial events in a human neuroblastoma cellular model (Figure 7a). Indeed, FGF1 inhibited etoposide-induced transactivation of the p53 target gene *PUMA,* ΔΨm decrease, caspase-9 and -3 activation and later events of apoptosis in the SH-SY5Y cell line either by an extra- or intracellular pathway (Figure 7b). It can be noticed that rFGF1 reduced etoposide-induced p53 phosphorylation, while *fgf1* overexpression did not. However, both extra- and intracellular FGF1 decreased p53-dependent transactivation of *PUMA*. These data suggest that extra- and intracellular FGF1 inhibit p53-dependent apoptosis by either decreasing p53 activation (extracellular FGF1) or by decreasing p53 transcriptional activity (extra- and intracellular FGF1). The difference between extra- and intracellular FGF1 pathways could be explained by the activation of the FGF receptors and its classical downstream signaling (PI3K/AKT and/or Ras/MAKP) by rFGF1. However, the mechanisms remain to be characterized.

The FGF1 anti-apoptotic activity was detected in human neuroblastoma SH-SY5Y cells, in rat neuron-like PC12 cells and in rat REtsAF fibroblast,^14, 15, 32, 33^ suggesting that this effect is conserved in different cell types and mammalian species. However, no activity of either extra- or intra-cellular FGF1 could be detected in the mouse neuroblastoma N2a cell line (Figures 7a and b), suggesting that this neuroblastoma-derived cell line was impaired in the FGF1 anti-apoptotic signaling pathway. It was previously shown that recombinant FGF2 activated both FGF receptors and MAPK in N2a cells.^34^ As FGF1 can bind and activate all FGF receptors, we hypothesize that in our study rFGF1 could also bind FGFR and activate the MAPK pathway in N2a cells and that the interference is either downstream of FGFR/MAPK activation or independent from MAPK.

The regulation of *fgf1* expression after rFGF1 or etoposide treatment also differed between the two neuroblastoma cell lines. In SH-SY5Y and PC12 cells, rFGF1 addition increased *fgf1* endogenous expression; this regulation was not detected in N2a cells (Figure 6a). In PC12 cells, transactivation of endogenous *fgf1* by exogenous FGF1 is a specific and important step for the neurotrophic activity of exogenous FGF1.^3^ Therefore, endogenous *fgf1* regulation by rFGF1 in both SH-SY5Y and PC12 cells correlates with rFGF1 anti-apoptotic activities, suggesting that endogenous *fgf1* expression regulation may be a crucial step for the anti-apoptotic activity of exogenous FGF1.

We also examined the regulation of *fgf1* expression in SH-SY5Y and N2a cells after etoposide treatment. We showed that etoposide treatment upregulated *fgf1* expression in SH-SY5Y cells, while downregulating *fgf1* expression in N2a cells; however, etoposide induced similar p53-dependent apoptosis in both cell lines. Sahu *et al.*^20^ described a similar p53-dependent apoptotic pathway in both neuroblastoma cell lines after treatment with the anticancer drug MPTQ. Therefore, our study favors the hypothesis that the difference in the regulation of *fgf1* expression by etoposide in both cell lines could be due to a difference in the mechanisms of *fgf1* expression regulation rather than a difference in the activated p53 pathways. Indeed, *fgf1* expression is mainly regulated at the transcriptional level by complex mechanisms in mammals. Different promoters, alternative splicing and multiple polyadenylation signals generate different *fgf1* transcripts (all coding for a single FGF1 protein). *Fgf1* expression is regulated in a tissue- and cell-specific manner, during development and at the adult stage.^1, 35, 36, 37, 38^ In human and mouse, promoter 1B is the major promoter in brain, gliomas and heart. Its activity is strongly linked to neural development and used to isolate neural stem/progenitor cells.^1, 39, 40, 41, 42^ We initiated the study of the regulation of alternative *fgf1* transcripts (1A to 1D) in SH-SY5Y and N2a cells and showed that: (i) the 1B *fgf1* mRNA is the major *fgf1* transcript in both cell lines, (ii) the regulation of the 1B *fgf1* mRNA by rFGF1 and etoposide is similar to the regulation of all *fgf1* transcripts, (iii) the other *fgf1* mRNAs could also be detected (1A and 1D in SH-SY5Y; 1A, 1C and 1D in N2a) and were regulated by rFGF1 and etoposide. Therefore, our study suggests that promoter 1B is the main promoter in human and murine neuroblastoma cell lines, according to previous studies showing its neuronal specificity,^1^ and must be further studied to progress in the characterization of the mechanisms involved in *fgf1* expression regulation in these cells. However, the other promoters (1A, 1C and 1D) could also be activated and regulated in these cells, showing the complexity of *fgf1* expression regulations. Their study should not be neglected.

We then investigated FGF1 intracellular activity in neuroblastoma cell lines and showed that overexpression of wild-type FGF1 protects SH-SY5Y cells from p53-dependent apoptosis while no protection was detected in N2a cells (Figure 7a). In SH-SY5Y cells, we further showed that overexpressed wild-type and mutant FGF1, which were not secreted in conditioned media, act mainly by an intracrine pathway in these cells. Both FGF1^WT^ and non-phosphorylable FGF1^S130A^ protected SH-SY5Y cells from p53-dependent apoptosis, in contrast to FGF1^K132E^ and FGF1^S130D^ (Figure 7c). Indeed, FGF1^WT^ and FGF1^S130A^ oppose genotoxic stress-induced *PUMA* transactivation, Delta;Ψm decrease, caspase activation and cell condensation. Both K132E and S130D mutations inhibit the FGF1 intracrine anti-apoptotic activity. This suggests that FGF1 C-terminal domain is important for the protein activity and that FGF1 phosphorylation inhibits its activity in SH-SY5Y cells, similarly to what we have previously shown in PC12 cells.^15^ FGF1 is a particular growth factor that contains a NLS mediating its nuclear translocation and it was shown by different laboratories (including ours) that FGF1 nuclearization is required for its mitogenic, neurotrophic and anti-apoptotic activity.^2, 14, 43^ In PC12 cells, our results suggested that FGF1 nuclear localization is required but not sufficient to mediate the FGF1 anti-apoptotic activity and that nuclear events regulate FGF1 activity.^15^ It was shown by Wiedlocha’s group that FGF1 is phosphorylated in the nuclear compartment by PKCδ and that FGF1 phosphorylation, which could be regulated by nucleolin, induced FGF1 nuclear export and degradation.^31, 44^ In PC12 cells, we detected no modification of the nuclear localization of the different FGF1 phosphorylation mutants compared to FGF1^WT^. By contrast, we showed that FGF1 phosphorylation impaired FGF1 anti-apoptotic activity, while it did not influence FGF1 neurotrophic activity. In SH-SY5Y cells, we showed that FGF1 phosphorylation also impaired its anti-apoptotic activity, suggesting that this regulation of intracrine FGF1 pathway is conserved in mammals. Since the anti-apoptotic and neurotrophic activities of nuclear FGF1 are probably mediated by FGF1-induced transcriptional regulations, the identification of the key genes involved in nuclear FGF1 activities in both PC12 and SH-SY5Y cells would be very informative to understand FGF1 signaling in relation to oncogenesis.

In neuroblastoma tumors and cell lines, very few studies previously examined FGF1 activity and expression.^13, 45, 46, 47^ In the present study, we showed that FGF1 inhibits p53-dependent apoptosis in SH-SY5Y cells by extracellular and intracellular pathways. In most primary human neuroblastomas, the oncosuppressor *p53* is not mutated and p53-dependent apoptosis can be activated by genotoxic stress or a specific activator, suggesting that upstream events impaired p53 activation in these tumors. In various human tumors such as ovarian cancer, astrocytomas and gliomas, *fgf1* overexpression is correlated with tumor progression and resistance to anti-tumor drugs.^5, 6, 7, 48^ Therefore, our data suggest that FGF1 may be involved in neuroblastoma tumor progression and chemoresistance. The study of FGF1 expression and activity must be pursued in a large panel of neuroblastoma tumors and cell lines.

In this paper, we show for the first time that a growth factor may act *via* an intracrine pathway to inhibit p53-dependent apoptosis in neuroblastoma. Similarly to FGF1, numerous growth factors and growth factor receptors can nuclearize and act by intracrine and nuclear pathways, whether in the FGF/FGFR family (FGF1-3,11-14/FGFR1)^2, 49, 50, 51^ or in other growth factor families such as EGF/EGFR, IGF-I/IGF-IR, VEGF/VEGFR.^52, 53, 54, 55, 56, 57^ In most of these studies, nuclearization of growth factors or of their receptors is associated with increased cell proliferation and survival and tumor chemoresistance. In human neuroblastoma, the expression of some of these factors or of their receptors was shown to be misregulated; however, potential intracrine and nuclear pathways of these proteins were not investigated. The study of these nuclear pathways in neuroblastoma must be performed and would probably allow the identification of new therapeutic targets.

## Materials and Methods

### Cell culture and drugs

SH-SY5Y cells are a subclone of the human neuroblastoma-derived cell line SK-N-SH,^58^ and were a kind gift from Dr. Alicia Torriglia. Neuro-2a (N2a) cells, a mouse neuroblastoma-derived cell line,^59^ were a kind gift from Dr. Véronique Dubreuil. Both cell lines were cultured in Dulbecco’s modified Eagle's medium supplemented with 10% fetal calf serum, 100 *μ*g/ml penicillin-streptomycin and 1% Glutamax at 37 °C in a humidified atmosphere of 5% CO_2_ (all reagents by Thermo Fisher, Waltham, MA, USA). At 50% confluence, 100 ng/ml human recombinant FGF1 (rFGF1) (R&D Systems, Minneapolis, MN, USA) and 10 *μ*g/ml Heparin (Sigma-Aldrich, St Louis, MO, USA) were added to media for 48 h before further treatment (rFGF1+heparin noted hereafter rFGF1 to simplify text and figures). When cells reached 70% confluence, 50 *μ*g/ml etoposide (E1383, Sigma-Aldrich) was added to the medium to induce p53-dependent apoptosis.

### FGF1 expression vectors

The wild-type human FGF1 coding region was subcloned into a constitutive mammalian expression vector (pcDNA3.1/V5-His, Invitrogen, Waltham, MA, USA) allowing the fusion with a C-terminal V5-His tag (pcDNA3.1-FGF1^WT^). Mutant FGF1 expression vectors were generated using the QuickChange II XL Site-Directed Mutagenesis Kit (Agilent Technologies, Santa Clara, CA, USA) according to the manufacturer’s protocol. We used the pcDNA3.1-FGF1^WT^ vector as a DNA template to generate pcDNA3.1-FGF1^S130A^, pcDNA3.1-FGF1^S130D^, pcDNA3.1-FGF1^K132E^ with specific primers. These vectors allow the expression of C-terminal V5-His tagged wild-type or mutant FGF1 (aa 15 to 154).

### Transfection

SH-SY5Y cells were stably transfected with 10 *μ*g of wild-type or mutant FGF1 expression vectors in the presence of 60 *μ*l of Lipofectin reagent (Invitrogen) in 100 mm Petri dishes as previously described.^3^ Geneticin-resistant (0.5 mg/ml, G418, Invitrogen) cells were selected over 4 weeks. Depending on the experiment, either monoclonal or polyclonal stable transfected cell lines were further analyzed.

For protein analysis, N2a cells were transiently transfected in 60 mm Petri dishes with either 1 *μ*g of pcDNA3.1-FGF1^WT^ or pcDNA3.1/V5-His (mock vector) in the presence of 9 *μ*l of Lipofectamine reagent (Invitrogen). For cell death analysis, N2a were co-transfected with pcDNA3.1-FGF1^WT^ or mock vector and with a GFP expression vector (pEGFP-N2, Clontech, Mountain View, CA, USA) in 12-well plates (0.2 *μ*g of each vector was transfected in the presence of 2 *μ*l of Lipofectamine reagent per well).

### Cell survival analysis by crystal violet nuclei staining assay

SH-SY5Y were plated in 12-well plates and treated with rFGF1 for 48 h. Etoposide was then added to induce p53-dependent apoptosis. Cell viability was estimated using the crystal violet method (0.1% crystal violet, 0.1 M citric acid) after 24 h of etoposide treatment as previously described.^33^

### Apoptosis analysis by flow cytometry assay

Adherent and non-adherent cells were harvested and double stained with PI(1:100, Sigma-Aldrich) and DiOC_6_(3) (1:5000, Molecular Probes, Eugene, OR, USA), or stained with CMX-Ros (1:1000, MitoTracker Red CMXRos, Life Technologies, Waltham, MA, USA).^33^ Flow cytometry analysis (PI, DIOC6(3), CMX-Ros, GFP staining and cell size analysis) were performed with a FACS LSR Fortessa (Becton-Dickinson, Franklin Lakes, NJ, USA) in the CYMAGES facility (UVSQ, Montigny-le-Bretonneux, France). DiOC_6_(3) staining allows to estimate the mitochondrial membrane potential (Delta;Ψm). A decrease of Delta;Ψm is characteristic of the mitochondrial pathway of apoptosis. Apoptosis is also characterized by both cell and nuclear condensation and plasma membrane integrity, in contrast to necrosis. PI staining allows to discriminate between necrotic cells (that is, primary and secondary necrosis are associated with plasma membrane permeabilization and thus PI^+^) and the viable and apoptotic cells (PI^−^). Apoptotic SH-SY5Y or N2a cells correspond to the cells with low PI and DiOC_6_(3) staining and low cell size. Apoptotic transfected N2a cells correspond to the GFP-positive cells with low CMX-Ros staining and low cell size.

### Western blot analysis

Adherent and non-adherent cells were collected and 20 *μ*g of proteins were separated in Mini-PROTEAN TGX 4–20% gels (Bio-Rad, Hercules, CA, USA) and transferred onto PVDF membranes (Millipore, Darmstadt, Germany). As previously described,^14^ blots were incubated in 5% milk-TBST (Tris Buffer Solution, 0.1% Tween-20, Sigma-Aldrich) with primary antibodies, washed in TBST and then incubated with appropriate HRP-conjugated secondary antibodies (Jackson, West Grove, PA, USA). Chemiluminescence was detected with a ChemiDoc XRS+ system; images were acquired and analyzed with the ImageLab software (Bio-Rad). The primary antibodies used in this study were: anti-FGF1 (AB-32-NA, R&D Systems), anti-His tag (A00186, GenScript, Piscataway, NJ, USA), anti-P-p53 (Ser-15) (9284S, Cell Signaling, Danvers, MA, USA), anti-PUMAα (N-19, Santa Cruz, Dallas, TX, USA), anti-Caspase-9 (5B4, Abcam, Cambridge, UK), anti-cleaved Caspase-3 (Asp175, Cell Signaling), anti-PARP (9542S, Cell Signaling) and anti-Actin (A2066, Sigma-Aldrich).

To test for the presence of FGF1^WT^, FGF1^S130A^ and FGF1^S130D^ in the conditioned media of SH-SY5Y transfected cells, FGF1 heparin-sepharose concentration was performed. Briefly, 120 *μ*g of total cell extract proteins or the equivalent fraction of conditioned media (about 1 ml) from transfected SH-SY5Y cell lines were incubated with 150 *μ*l of heparin-sepharose (CL-6B, GE Healthcare, Little Chalfont, UK) in PBS containing 0.66 M NaCl and protease inhibitors (cOmplete Mini, EDTA-free, 1:100, Roche, Basel, Switzerland). After an overnight fixation at 4 °C, the heparin-sepharose was washed thrice with binding buffer, and the heparin-binding proteins were eluted in 60 *μ*l NuPage LDS sample buffer 2 × (Life technologies) containing 100 mM DTT at 96 °C for 10 min.

### FGF1 mRNA analysis by RT-PCR assay

SH-SY5Y and N2a cells were plated in 100 mm Petri dishes and treated with rFGF1 for 72 h or with etoposide for 16 h. Total RNAs were extracted using the RNA Nucleospin kit (Macherey-Nagel, Düren, Germany) according to the manufacturer’s protocol. RT-PCR was performed to examine FGF1 mRNA and 18S rRNA as previously described.^3^ Amplified products were separated in 8% acrylamide gels, stained with ethidium bromide, photographed with a GeneStore system (SynGene, Cambridge, UK) and quantified using the ImageQuant software (GE Healthcare). The primers used for RT-PCR amplification of all human and mouse *fgf1* mRNAs are complementary to *fgf1* coding sequences, which are present in all *fgf1* mRNAs: the forward FGF1 primer 5′-AAGCCCGTCGGTGTCCATGG-3′ and the reverse FGF1 primer 5′-GATGGCACAGTGGATGGGAC-3′. The primers used for RT-PCR of specific amplification of *fgf1* mRNAs (1A to 1D) in human and mouse are complementary to alternative 5′UTRs and coding *fgf1* sequences (as described in supplementary Table 1). We performed 18S rRNA RT-PCR as control and for quantification using the following primers: a forward 18S primer 5′-GTAACCCGTTGAACCCCATT-3′ and a reverse 18S primer 5′-CCATCCAATCGGTAGTAGCG-3′.

### Statistical analysis

The values presented in the different graphs indicate the mean and standard error of the mean (S.E.M.) for at least three independent experiments. Two-tailed unpaired Student’s *t*-tests were performed.
