# Supplementary material for: FGF1 protects neuroblastoma SH-SY5Y cells from p53-dependent apoptosis through an intracrine pathway regulated by FGF1 phosphorylation
Source: Cell Death Dis. 2017 Aug 31;8(8):e3023–. doi: 10.1038/cddis.2017.404 (PMC5596585; doi:10.1038/cddis.2017.404)
Supplement: Supplementary Figure 1 [file cddis2017404x1.pdf]

Supplementary Figure 1 - Regulation of alternative 1A, 1B, 1C and 1D *fgf1* mRNA levels by rFGF1 or etoposide in SH-SY5Y and N2a cells

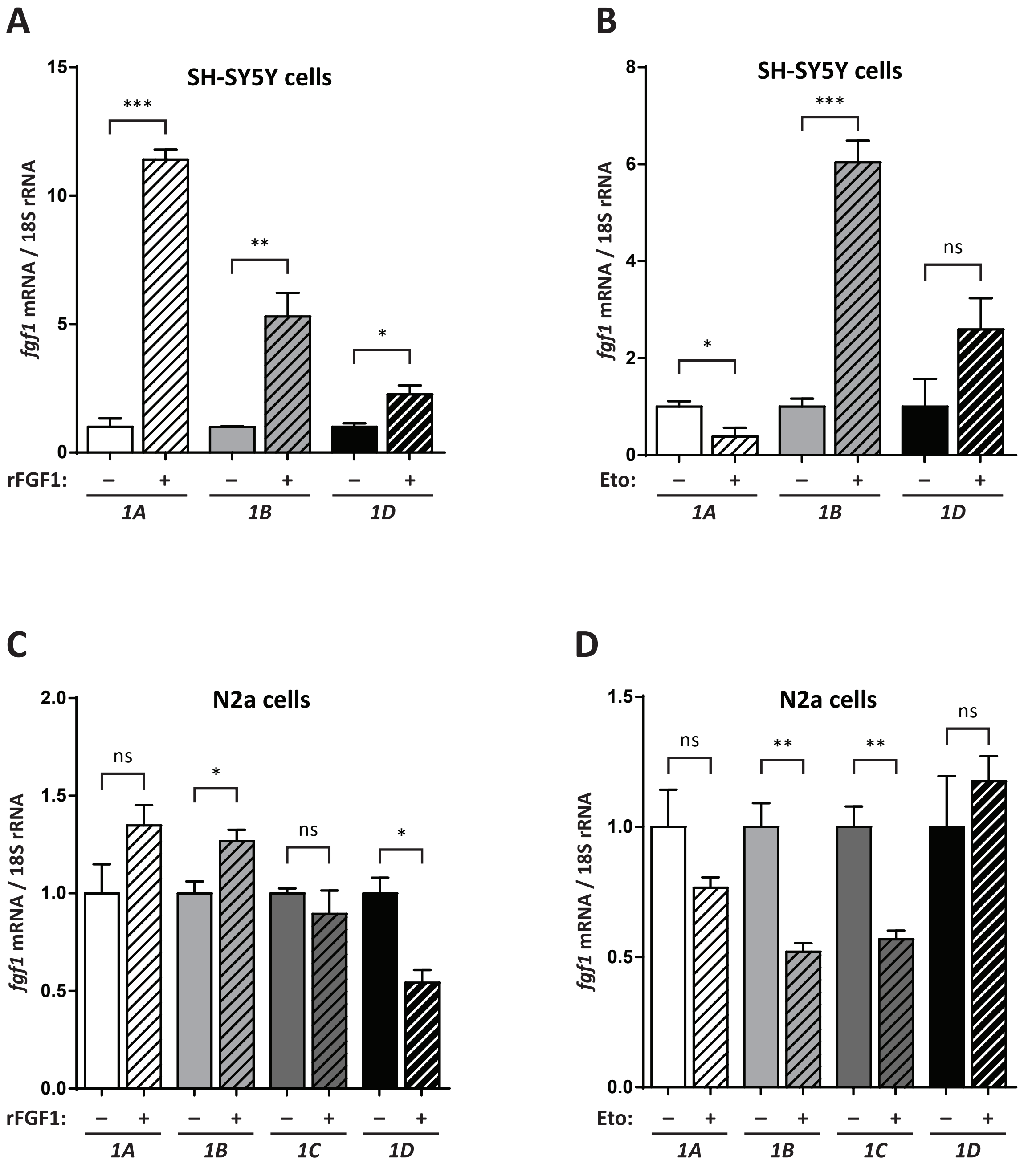

SH-SY5Y and N2a cells were treated or not with rFGF1 for 72h (**A-C**) or etoposide for 16h (**B-D**). Alternative 1A, 1B, 1C and 1D *fgf1* mRNA levels were analyzed by RT-PCR using specific primers described in supplementary Table 1. The 18S rRNA levels were used as a control for quantifications. The graphs represent the mean +/- SEM of three independent experiments. Student's *t*-tests were performed (n=3; ns: $p>0.05$; *: $p\leq 0.05$; **: $p\leq 0.01$; ***: $p\leq 0.001$).
