# Supplementary material for: FGF1 protects neuroblastoma SH-SY5Y cells from p53-dependent apoptosis through an intracrine pathway regulated by FGF1 phosphorylation
Source: Cell Death Dis. 2017 Aug 31;8(8):e3023–. doi: 10.1038/cddis.2017.404 (PMC5596585; doi:10.1038/cddis.2017.404)
Supplement: Supplementary Table 1 [file cddis2017404x2.pdf]

Supplementary table 1 – Specific primers used for RT-PCR of alternative 1A, 1B, 1C and 1D *fgf1* mRNA

Primers used in murine N2a cell line

Primer (5' → 3')	Sequence	Size of amplified fragment (bp)	Number of cycles
**Mouse *fgf1* Reverse (Exon 1)***	TGTGATCTCCCCTTCAGCCAT	*Used for all amplifications*	
**Mouse *fgf1-1A* Forward**	CTGCCACCACCCAGACAGGA	92	33
**Mouse *fgf1-1B* Forward**	CGGACTTCATTCCCGTCTTGT	121	28
**Mouse *fgf1-1C* Forward**	GAAGCAGGCACCAGGCAGAG	164	35
**Mouse *fgf1-1D* Forward**	TGGATGCAGGATTTCCAAAG	196	35

Primers used in human SH-SY5Y cell line

Primer (5' → 3')	Sequence	Size of amplified fragment (bp)	Number of cycles
**Human *fgf1* Reverse (Exon 1)***	GGTGATTTCCCCTTCAGCCAT	*Used for all amplifications*	
**Human *fgf1-1A* Forward**	AATCAGGGCATCGCCTCCTTT	223	46
**Human *fgf1-1B* Forward**	CACTCAGAGCTGCAGTAGCCT	194	37
**Human *fgf1-1C* Forward**	ACTTCTGCAGGGAAGCCAGC	88	–
**Human *fgf1-1D* Forward**	AGCAGCACAATGTTTGGGCTA	84	40

* Reverse primers (human or murine) located in *fgf1* exon 1 were used for all amplifications in combination with one of four specific forward primers. The size of the resulting amplified fragments and the number of amplification cycles needed to detect them are indicated.
